# The efficacy of gemcitabine and docetaxel chemotherapy for the treatment of relapsed and refractory osteosarcoma: A systematic review and pre‐clinical study

**DOI:** 10.1002/cam4.70248

**Published:** 2024-09-24

**Authors:** Kaan Low, Paola Foulkes, Frank Hills, Helen C. Roberts, Britta Stordal

**Affiliations:** ^1^ Department of Natural Sciences Middlesex University London UK

**Keywords:** docetaxel, gemcitabine, relapsed osteosarcoma

## Abstract

**Introduction:**

Osteosarcoma is the most common primary malignancy of the bone. There is a lack of effective treatments for patients who experience relapsed osteosarcoma. One treatment for relapsed patients is gemcitabine and docetaxel combination chemotherapy (GEMDOX). This systematic review aimed to establish the efficacy of this chemotherapy regimen, as well as identify the common severe toxicities that are associated with it. Resistant osteosarcoma cell lines developed from MG‐63 and HOS‐143B were used to represent relapsed osteosarcoma patients in a pre‐clinical study.

**Results:**

We identified 11 retrospective and Phase II studies that were suitable for inclusion in our review. 10.65% of patients had a response to gemcitabine and docetaxel combination therapy and the disease control rate was 35% (*n* = 197). 36%, 35.3% and 18.04% of patients experienced grade 3 or 4 neutropenia, thrombocytopenia and anaemia respectively (*n* = 133). Male patients (*X* ^2^ = 9.14, *p* < 0.05) and those below the age of 18 (*X* ^2^ = 10.94, *p* < 0.05) responded better to GEMDOX treatment than females and patients older than 18 years. The resistant osteosarcoma cell lines remained sensitive to either single‐agent gemcitabine, docetaxel, and the combination of both. Cisplatin‐resistant models (MG‐63/CISR8 & HOS‐143B/CISR8) were the most responsive to GEMDOX treatment compared to doxorubicin, methotrexate, and triple‐combination resistant models.

**Conclusion:**

GEMDOX treatment has potential efficacy in relapsed osteosarcoma patients especially those with cisplatin resistance. To directly compare the efficacy of GEMDOX therapy against other therapies randomised phase III clinical trials with adequate patient follow up must be performed to improve treatment options for osteosarcoma.

## 1 INTRODUCTION

Osteosarcoma is a primary bone malignancy that arises from the mesenchymal stem cells of the bone marrow.^1^ The overall 5‐year survival rate of osteosarcoma patients is approximately 60%–70%.^1^, ^2^, ^3^ However, approximately 40% of patients with non‐metastatic osteosarcoma will relapse, with the average 5‐year survival rate reducing to 30%.^4^ The presence of metastasis is also an adverse prognostic factor and patients who present with metastasis at the time of diagnosis only have a 5‐year survival rate of up to 30%.^2^, ^4^

The current standard frontline chemotherapy treatment for osteosarcoma patients includes the combination of methotrexate, doxorubicin and cisplatin (MAP).^5^, ^6^ The high rate of disease recurrence in osteosarcoma patients highlights the need for clear guidance on therapies for the treatment in the recurrent setting. Several agents have been explored for recurrent osteosarcoma including etoposide, interferon α‐2b and sorafenib.^7^ One of the most documented alternative chemotherapy regimens is GEMDOX, the combination of gemcitabine and docetaxel.^7^ GEMDOX therapy has been demonstrated to be an effective treatment for other sarcomas, especially leiomyosarcomas.^8^, ^9^ A retrospective study conducted by Leu et al. examined the efficacy of GEMDOX therapy in sarcomas patients (including osteosarcoma) demonstrated an overall response rate of 43%, and a disease control rate of 80%.^10^

Due to the relatively small number of osteosarcoma patients diagnosed each year, only one prospective Phase II clinical trial investigating GEMDOX therapy as a second‐line treatment for osteosarcoma patients has been conducted.^11^ However, several institutions have retrospectively reviewed the outcomes of osteosarcoma patients who received GEMDOX combination therapy for recurrent disease. The promising outcomes for sarcoma patients receiving GEMDOX therapy,^10^ along with a relative abundance of retrospective osteosarcoma patient data, resulted in GEMDOX therapy being a timely topic for a systematic review study.

Common side effects that are associated with docetaxel monotherapy include neutropenia, hypersensitivity, oedema and peripheral neuropathy.^12^, ^13^ Gemcitabine has some severe side effects associated with its administration, which include skin reactions, oedema and myelosuppression.^14^ The most severe side effects associated with gemcitabine and docetaxel combination therapy are haematological, as a result of myelosuppression.^15^

In addition, a panel of pre‐established chemoresistant osteosarcoma cell lines developed with frontline MAP regimen^16^ were used in this study to investigate the sensitivity of these resistant models to GEMDOX therapy. The panel of resistant cell lines established from MG‐63 and HOS‐143B includes models developed by using single‐agent of cisplatin, doxorubicin or methotrexate and the combination of three (multi‐agent). These chemoresistant models are used to examine the efficacy of GEMDOX therapy in vitro as they could represent the relapsed and refractory osteosarcoma patients in a standard clinical setting who received a frontline MAP regimen.

In this study, we first explore the efficacy and toxicity of GEMDOX combination chemotherapy as a second‐line treatment for relapsed and refractory osteosarcoma through a systematic review and meta‐analysis of published studies. We aimed to determine the efficacy of GEMDOX combination treatment for relapsed osteosarcoma patients and to determine if the efficacy was associated with the characteristics of patients such as age, sex and drug doses administrated for gemcitabine. Furthermore, we also examine GEMDOX treatment on chemoresistant osteosarcoma cell lines that model relapsed and refractory osteosarcoma patients.

## 2 MATERIALS AND METHODS

### 2.1 Systematic review

#### 2.1.1 Identification of relevant studies

Searching for relevant literature was conducted up to 30 November 2023 using PubMed (http://www.ncbi.nlm.nih.gov/pubmed). The search terms in the search strategy included as follows: ‘Osteosarcoma OR osteogenic sarcoma OR bone cancer OR bone sarcoma’, ‘Second‐line OR refractory OR recurrent OR resistant OR relapsed’, ‘Gemcitabine’, ‘Docetaxel OR Taxotere OR DTX OR GEMDOX’. Overlapping studies from the same authors were excluded. Due to the limited number of Phase II trials for osteosarcoma patients, retrospective studies were included, and the search was not restricted to a specific time.

#### 2.1.2 Inclusion and exclusion criteria

Studies were included according to the following criteria: (1) patients of any age had to have been diagnosed with osteosarcoma (papers that studied other sarcomas were acceptable only if the outcome data for osteosarcoma patients was extractable); (2) patient's osteosarcoma had to be relapsed or refractory (metastatic or non‐metastatic); (3) patients had to have received a previous chemotherapy regimen for their disease (single‐agent cisplatin or MAP therapy); (4) any prior chemotherapy regimen had not included gemcitabine or docetaxel; (5) patients had to be receiving gemcitabine and docetaxel as combination therapy, not either as a single‐agent therapy. Studies were excluded according to the following criteria: (1) the clinical trial had not been conducted; (2) patient treatment history was unavailable; (3) Data for osteosarcoma patients could not be extracted.

#### 2.1.3 Quality assessment

To assess the quality of the retrospective studies, a set of five questions was compiled from different quality assessment tools including the NIH Study Quality Assessment Tool for Cohort Studies^17^ and the Critical Appraisal Skills Programme checklist for cohort studies.^18^ The questions were as follows: (1) were the participants a representative sample of the target population; (2) were there clearly defined inclusion and exclusion criteria; (3) were the outcomes of interest and length of follow up clearly defined; (4) were the reasons for discontinuation of treatment and loss to follow up documented; (5) was valid statistical analysis of the data performed. The quality of each paper was assessed by two independent researchers (K.L., P.F.), any disagreements were resolved by a third party (B.S.).

#### 2.1.4 Data collection and analysis

The review site Covidence was used to collate the data extracted from each paper. Data was extracted according to a purpose‐built extraction template in Covidence (https://app.covidence.org). All data was extracted by two independent researchers (K.L., P.F.) and any disagreements were resolved by a third party (B.S.). Data collected from the papers included the primary author, author contact details and the institution where the research was carried out. Patient characteristics included age, sex and previous treatments. Intervention details included the dose of docetaxel and gemcitabine patients received, the administration schedule, the number of cycles and any additional medications that were given alongside treatment.

The primary outcomes that were extracted from the studies include overall survival (OS), progression‐free survival (PFS) and tumour response. The number of patients who experienced a complete response (CR), partial response (PR), stable disease (SD) or progressive disease (PD) were also collected. These measures were defined by Response Evaluation Criteria in Solid Tumour (RECIST) for all papers.^19^ A CR is defined as the ‘disappearance of all target lesions’, whilst a PR is characterised by a minimum 30% decrease in the overall diameter of all target lesions.^19^ Progressive disease is defined as a minimum 20% increase in the sum of the diameters of the target lesions, as well as the appearance of one or more new lesions.^19^ Finally, SD refers to incidence where the shrinkage or increase in lesion diameter is insufficient to classify as PR or progressive disease.^19^ All response data was extracted as raw count data from each study, pooled and then percentage data compared across subgroups.

The secondary outcomes extracted were the grade 3 or 4 haematological and neuropathological toxicities including neutropenia, thrombocytopenia, anaemia and neuropathy were recorded, along with any instances where treatment was discontinued due to toxicity. Any pre‐treatment medications that patients received to ameliorate or prevent toxicity were also recorded. All studies included in this review used the common terminology criteria for adverse events (CTCAE) to assess toxicities.^20^ Grade 3 toxicities are considered to be ‘severe or medically significant’ and require hospitalisation, whilst grade 4 toxicities have ‘life‐threatening consequences’ and require urgent treatment.^20^ All toxicity data was extracted as raw count data from each study, pooled and then percentage data compared across subgroups.

The data collected were summarised according to patient characteristics, response data, toxicity data, dose data and survival data, with availability of each data set indicated for each study. Any data that was unavailable due to a lack of reporting or unextractable from the wider data set was indicated by colour coding.

### 2.2 In vitro studies

#### 2.2.1 Cell culture

Human osteosarcoma cell lines MG‐63 and HOS‐143B were sourced from University College London. Cells were grown in DMEM (Gibco, Thermo Fisher Scientific) supplemented with 10% foetal calf serum (Gibco, Thermo Fisher Scientific), 1% sodium pyruvate (Gibco, Thermo Fisher Scientific), 1% non‐essential amino acids (NEAA) (Gibco, Thermo Fisher Scientific), free of antibiotics. All cells were maintained in a humidified atmosphere of 5% CO2 at 37°C. Only cells at log phase of growth were used in the experimentations. Cell lines were routinely checked for mycoplasma and were mycoplasma‐free.^21^

Resistant models were developed from MG‐63 and HOS‐143B by either introducing single‐agent or multi‐agent of chemotherapeutic drugs that are commonly used in the standard first‐line chemotherapy treatment for osteosarcoma patients (cisplatin, doxorubicin and methotrexate).^16^ MG‐63/CISR8 and HOS‐143B/CISR8 are generated by single‐agent cisplatin; MG‐63/DOXR8 and HOS‐143B/DOXR8 by single‐agent doxorubicin; MG‐63/MTXR8 and HOS‐143B/MTXR8 by single‐agent methotrexate; and MG‐63/TRIR8 and HOS‐143B/TRIR8 by multi‐agent of cisplatin, doxorubicin and methotrexate. All resistant models were cultured under the similar conditions as the parental MG‐63 and HOS‐143B cell lines.

#### 2.2.2 Cytotoxicity assay

The sensitivity of the cells to chemotherapy drugs was determined by acid phosphatase assay. Cells were plated into 96‐well plates at the cell density of 1 × 10^4^ cells/well and the cells were allowed to attach overnight. Serial dilutions of gemcitabine (Sigma–Aldrich) and docetaxel (Sigma–Aldrich) were used to treat the wells in triplicate in a final volume of 200 μL. The highest drug concentration used for gemcitabine was 8 ng/mL, docetaxel was 20 ng/mL. The combination was optimised from the highest drug concentration used in single‐agent cytotoxicity assay. The final highest concentrations used for the GEMDOX (combination of gemcitabine and docetaxel) was gemcitabine at 0.8 ng/mL and docetaxel at 2 ng/mL. Drug‐free controls were added with 100 μL of fresh growth medium. Cells were then incubated for 5 days at 37°C in 5% CO_2_ and an acid phosphatase assay was used to determine cell viability.^22^ On Day 5, the media was discarded from the wells and washed twice with PBS. Concentration of 2.63 mg/mL of phosphatase substrate (Sigma–Aldrich) was dissolved in sodium acetate buffer and added 100 μL to each well. After incubating the plate at 37°C for 1 h, 50 μL of 1 M sodium hydroxide was added and absorbance was measured at 405 nm on the plate reader (Omega FLUOStar, BMG Labtech).

### 2.3 Statistical analysis

#### 2.3.1 Systematic review

A weighted average of median participant age was produced in SPSS; median age was weighted by the number of participants to account for the different patient numbers in each study. Subgroups of studies were created according to the following characteristics: the median age of the participants (<18 or ≥18), the dose of gemcitabine that patients received (675 or 1000 mg/m^2^) and their sex (studies that contained approximately equal numbers of males and females, and studies that contained approximately double the number of males than females). A Chi‐square test of association was used to compare the frequencies of categorical variables, including response data and toxicity data. Comparisons were performed between the two subsets of studies in the subgroups of age, dose of gemcitabine and sex. Statistical significance was defined as a *p* value ≤ 0.05.

#### 2.3.2 In vitro studies

All experiments were repeated at a minimum in biological triplicate. Statistical significance analysis was performed by two‐sample *t*‐test analysis in Minitab (version 19.2020.1.0) using a two‐tailed distribution and two samples of equal variance settings. Graphs were made by using GraphPad Prism (version 8.4.1; GraphPad Software, La Jolla, CA, USA). *p* < 0.05 was considered to indicate a statistically significant difference.

## 3 RESULTS

### 3.1 Systematic review—Eligible studies

**FIGURE 1:**
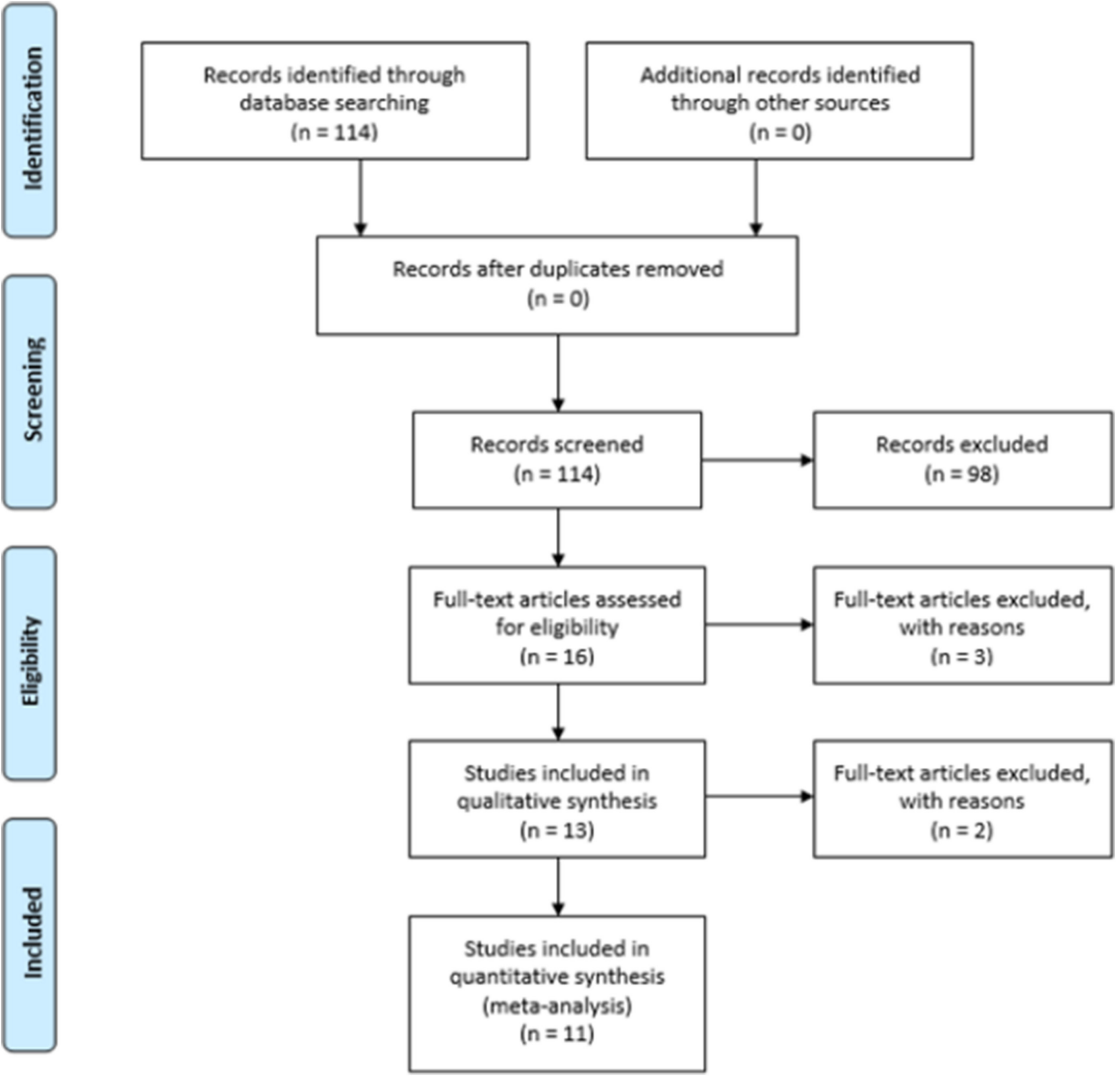
PRISMA flow diagram detailing the search and selection process employed during the systematic literature search and review.

The total number of results produced by the PubMed search strategy was 114. After screening the abstracts for suitability, 98 papers were excluded. Next, the remaining 16 full texts were assessed. Thirteen papers were retained for data extraction (Figure 1). Following data extraction, two pairs of studies^15^, ^23^, ^24^, ^25^ were determined to have been carried out at the same institutions with overlapping patient enrolment periods. The papers were reviewed and one paper from each institution was excluded to avoid the duplication of patient data, as it was not possible to identify which patients had been included in both studies. The two studies selected for inclusion^24^, ^25^ had more recent publication dates, a longer study duration and a larger number of participants.

### 3.2 Characteristics and summary of included studies

Eleven studies with a total of 197 evaluable patients met the inclusion criteria for this review (Figure 1). Ten of the studies were retrospective reviews,^24^, ^25^, ^26^, ^27^, ^28^, ^29^, ^30^, ^31^, ^32^, ^33^ whilst one study^11^ was a Phase II single‐arm study. No data was available for the number of previous cycles of chemotherapy that patients received, or their grade of cancer. Due to the loss of follow‐up and the inclusion of patients with different types of sarcomas, not all data was extractable for all studies. Patient characteristics including weighted mean age, sex and histology for all included patients in the review are summarised in Table 1. In our quality assessment, none of the studies had scored highly indicating a risk of bias for any of the five questions. Four of the 11 studies determined to have a low risk of bias across the five questions.^25^, ^27^, ^30^, ^33^ The remaining seven studies scored either a low risk or unclear risk of bias.^11^, ^24^, ^26^, ^28^, ^29^, ^31^, ^32^ In overall, the risk of bias for all the eligible studies included in this review was low (Table S1).

**TABLE 1:** Summary of the patient characteristics included in the review. Median age was weighted by the number of participants in each study.

	All participants	Data availability (%)
No. of participants	197	100
Weighted mean age	18.37 years	100
Sex	67 males and 45 females	57
Histology	Conventional–93% Other–7%	50

**FIGURE 2:**
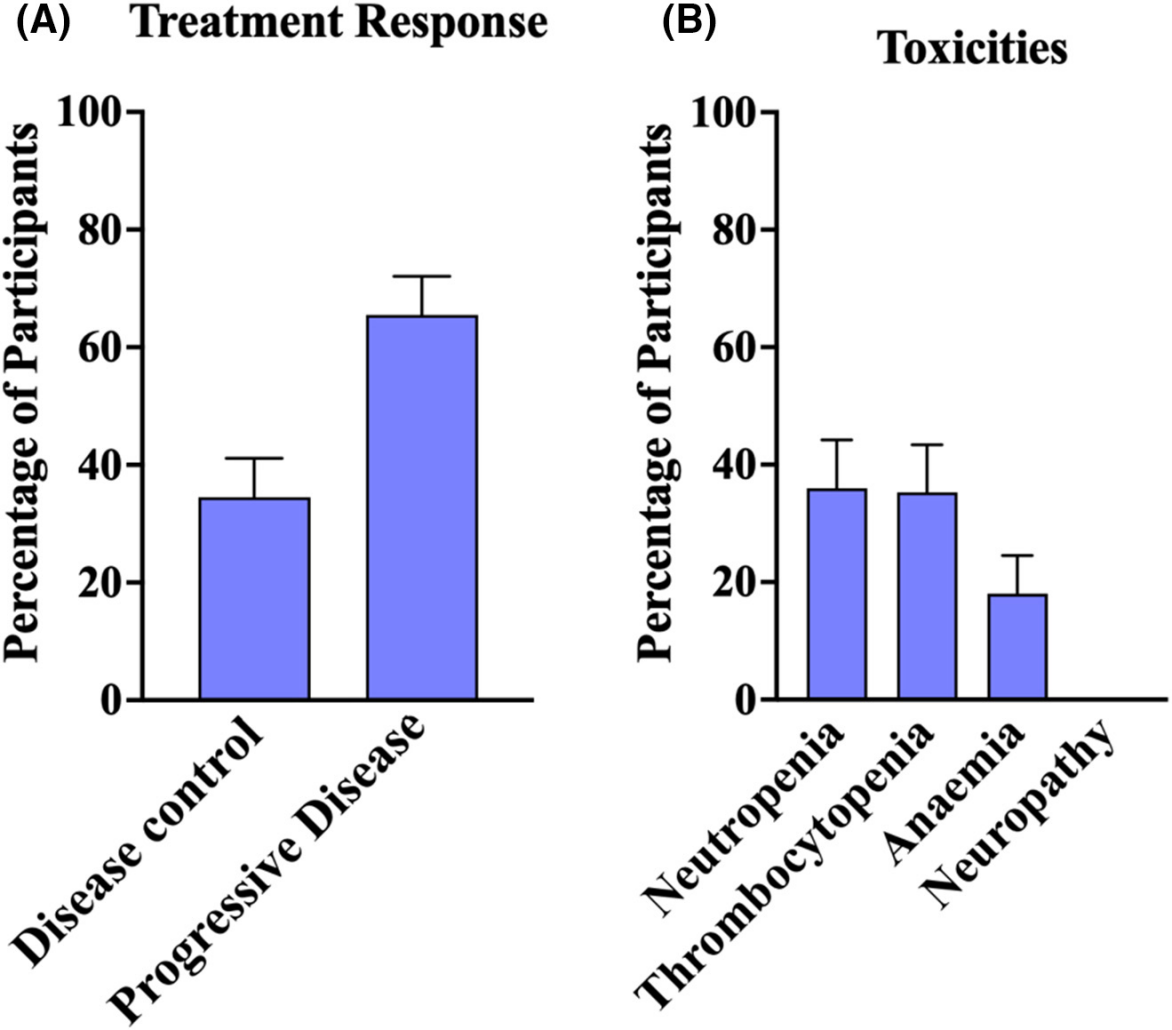
Disease control and toxicity outcomes of GEMDOX treatment. (A) Percentage of the patients included in the review whose disease was controlled with GEMDOX treatment. Disease control is defined as patients who experience CR, PR or SD. (B) Incidence of Grade 3–4 toxicities during GEMDOX treatment. Data are presented as percentage + 95% CI (*n* = 197).

The doses of gemcitabine and docetaxel that were administered in each study and the patient response outcomes across all studies were collated in Covidence and summarised in Table 2. Out of all 197 patients, four patients (2.03%, 95% CI 0.1%–4%) experienced a CR to treatment, 17 patients (8.63%, 95% CI 4.7%–12.6%) of patients experienced a PR, 47 patients (23.86%, 95% CI 17.9%–29.8%) of patients had SD and 129 patients (65.48%, 95% CI 58.8%–72.1%) experienced disease progression (Table 2). Overall, the proportion of patients who responded to GEMDOX treatment (experiencing either a CR, PR or SD) was 34.52% (95% CI 27.9%–41.2%), whilst the proportion of patients who did not respond to GEMDOX treatment (experienced progressive disease) was 65.48% (95% CI 58.8%–72.1%) (Figure 2A).

**TABLE 2:** Summary of the dose and response data for the 197 patients included in the review.

Author/year	Study design	No. of patients	Median age	Males	Dosage used		Treatment response			
					Gemcitabine (mg/m^2^)	Docetaxel (mg/m^2^)	Complete response	Partial response	Stable disease	Progressive disease
Fox et al., 2012^11^	P	14	36.2	43%	675	75	0	1	3	10
Gosiengfiao et al., 2012^26^	R	2	16	100%	675	75/100	0	1	1	0
He et al., 2013^27^	R	23	18	65%	1000	75	0	3	8	12
Lee et al., 2016^24^	R	28	15.7	NE	675/900	100	3	1	4	20
Mora et al., 2009^28^	R	1	14	NE	1000	100	0	0	1	0
Navid et al., 2008^29^	R	10	NE	NE	675	75/100	0	3	1	6
Palmerini et al., 2016^30^	R	35	14.5	NE	675/900	75	0	6	14	15
Rapkin et al., 2012^31^	R	6	14.5	NE	675	75	1	0	2	3
Takahashi et al., 2017^32^	R	5	NE	NE	900	70	0	0	4	1
Xu et al., 2018^33^	R	52	18.4	56%	1000	75	0	0	5	47
Yu et al., 2014^25^	R	21	NE	71%	675	75	0	2	4	15
							2.03%	8.63%	23.86%	65.48%

Abbreviations: NE, not extractable from study; P, prospective; R, retrospective.

Data for the following grade 3 and 4 toxicities were summarised: neutropenia, thrombocytopenia, anaemia and neuropathy. Any incidence of discontinuation due to treatment toxicity was also included. Across all the studies, only 2% of patients discontinued GEMDOX treatment due to treatment toxicity. Toxicity data was only available for 133 of the 197 patients. Grade 3–4 neutropenia and thrombocytopenia occurred in 36.1% (95% CI 27.9%–44.3%) and 35.3% (95% CI 27.2%–43.5%) of patients respectively, whilst grade 3–4 anaemia occurred in 18.04% (95% CI 11.5%–24.6%) of patients. No studies reported any incidence of severe neuropathy (Figure 2B).

Complete survival data was available for five of the 11 studies. The median PFS for the studies ranged from 1 to 3 months, with three individual patients presenting with a duration of response that lasted a minimum of 12 months. The median OS ranged from 6 to 9 months, with the longest documented survival of an individual patient of being 69 months.^29^ Sixteen patients from Lee et al.'s study were still alive 1 year after receiving GEMDOX treatment. Due to the variety of reporting measures employed by the researchers, we were unable to perform statistical analysis on the survival data.

### 3.3 Response to GEMDOX treatment is dependent on Age but not Gemcitabine Dose

**FIGURE 3:**
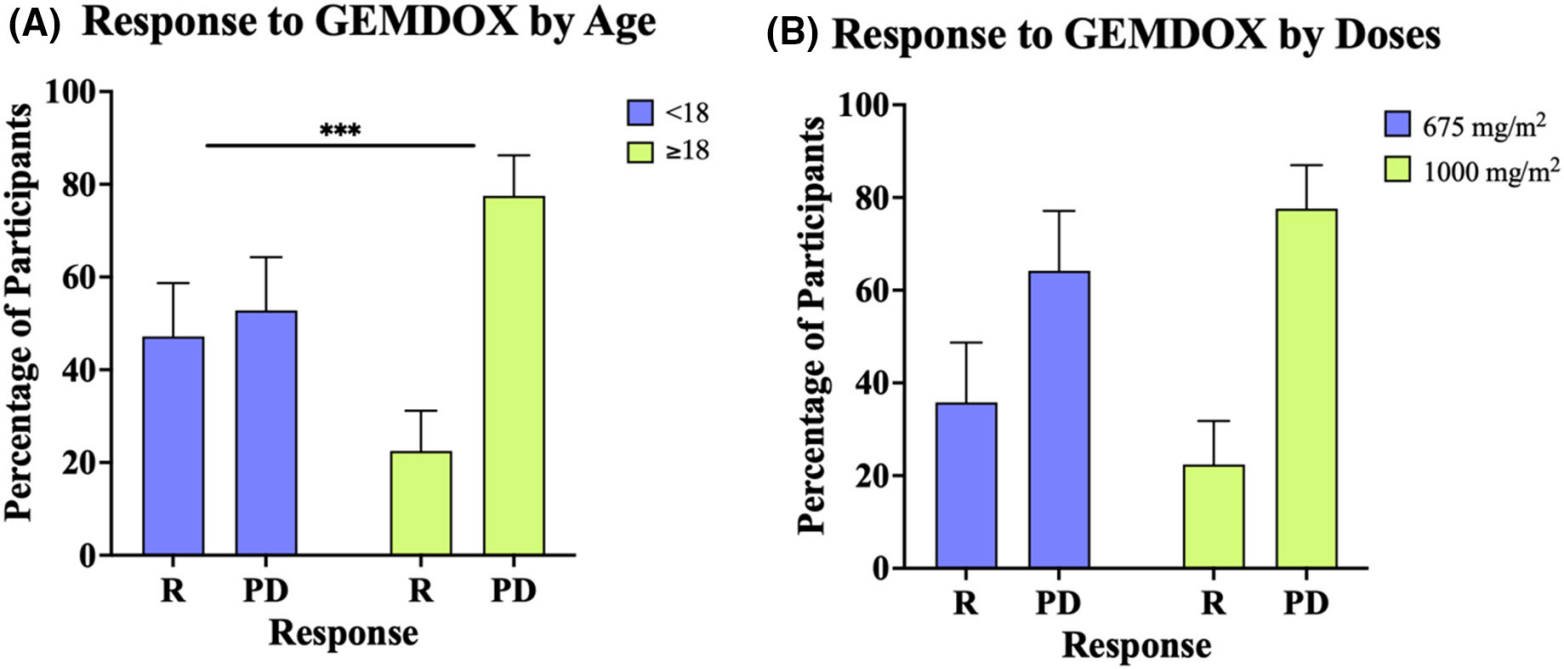
Disease Control to combination of GEMDOX regimen by (A) age, and (B) dose of gemcitabine. Percentage of participants on y‐axis and the response of the participants on x‐axis. *R* represents participants who responded to the treatment (complete response, partial response and stable disease) and PD represents not responded (progressive disease). Data are presented as percentage + 95% CI, ***p < 0.001, *X* ^2^ Age *n* = 161; Dose *n* = 88.

Five of the studies^24^, ^26^, ^28^, ^30^, ^31^ had a median participant age of <18. Whereas, three studies had a median participant age of ≥18 years (Table 2)^11^, ^27^, ^33^ The response data for all participants in these two age groups were pooled to produce a total count of participants whose disease was controlled by GEMDOX treatment (CR + PR + SD) and a total count of participants with progressive disease. Disease control in response to GEMDOX treatment was determined to be dependent on the age of participants, 47.2% (95% CI 35.7%–58.8%) of patients from the studies with a median age of <18 responded to GEMDOX treatment compared to 22.47% (95% CI 13.8%–31.1%) of patients from the papers with a median age of ≥18, *X* ^2^ (1, *N* = 161) = 10.94, *p* < 0.05 (Figure 3A).

Across all 11 studies included in this review, three different doses of gemcitabine were administered: 675, 900 or 1000 mg/m^2^ (Table 2). Only one study^32^ administered gemcitabine at a dose of 900 mg/m^2^. Three papers included in this review^27^, ^28^, ^33^ administered gemcitabine at a dose of 1000 mg/m^2^. Five papers^11^, ^25^, ^26^, ^29^, ^31^ administered gemcitabine at a dose of 675 mg/m^2^ (Table 2). As the number of patients treated with 900 mg/m^2^ of gemcitabine was insufficient for Chi‐square analysis, we decided to compare the response data for patients treated with 675 or 1000 mg/m^2^ of gemcitabine. Chi‐square analysis determined no significant association between the dose of gemcitabine and the patients' response to GEMDOX treatment, *X* ^2^ (1, *N* = 88) = 1.41, *p* > 0.05 (Figure 3B).

### 3.4 The incidence of toxicity is not dependent on age and gemcitabine dose

**FIGURE 4:**
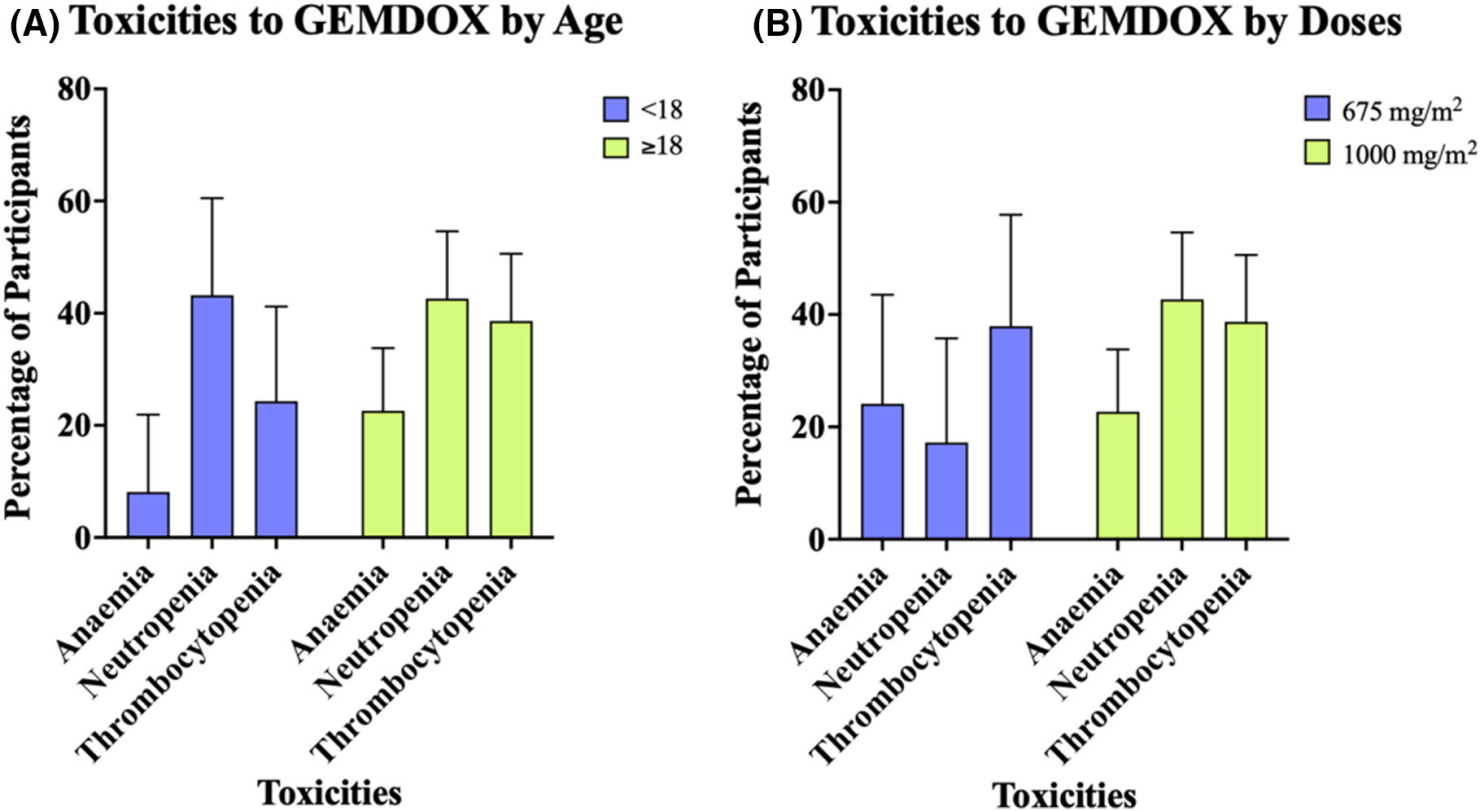
(A) Percentage of patients who <18 or ≥18 who experienced grade 3 or 4 toxicities. (B) Percentage of patients who received 675 or 1000 mg/m^2^ of gemcitabine who experienced grade 3 or 4 toxicities. Data are presented as percentage + 95% CI, *p* > 0.05, *X* ^2^ Age *n* = 105 and Dose *n* = 101.

Across the seven studies for which toxicity data was available, there were 119 cases of grade 3 or 4 haematological toxicities. To determine whether incidence of grade 3–4 toxicity was associated with the age of participants, two of the three studies with a median participant age ≥18 had toxicity data available.^27^, ^33^ Each of the four toxicities were analysed independently. The total counts for each of the recorded toxicities was calculated for each age group and a Chi‐square test of association was performed to determine whether there was an association between the age of the participants and the incidence of grade 3–4 toxicities. No significant association was found between the age of the participants and the incidence of grade 3–4 toxicities, *X* ^*2*^ (2, *N* = 105) = 3.84, *p* > 0.05 (Figure 4A). Of the studies where patients had received gemcitabine at a dose of 1000 mg/m^2^ and 675 mg/m^2^, 2 and 3 studies respectively had toxicity data available. Chi‐square analysis determined no significant association between the dose of gemcitabine and the incidence of grade 3–4 toxicities, *X* ^2^ (2, *N* = 101) = 2.87, *p* > 0.05 (Figure 4B).

### 3.5 In vitro study

#### 3.5.1 Sensitivity profile of gemcitabine and docetaxel

**FIGURE 5:**
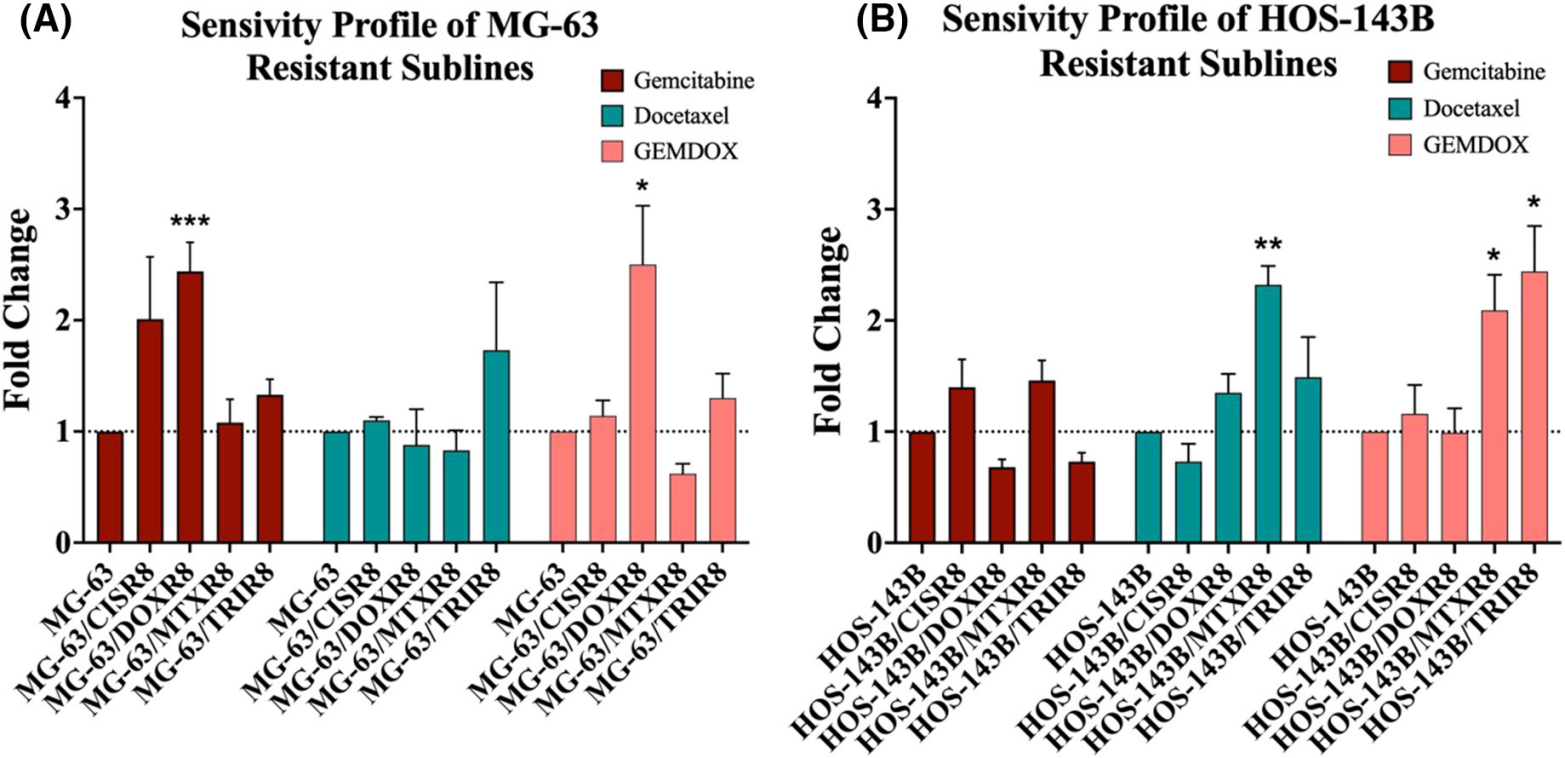
Cytotoxicity assays were performed to determine the fold change of the gemcitabine, docetaxel and the combination of both. Fold resistance of gemcitabine, docetaxel and GEMDOX combination treatment on (A) MG‐63 resistant sublines and (B) HOS‐143B resistant sublines comparing to their parental cell lines. Error bars represent SEM (*n* = 3). *p < 0.05, **p < 0.01, ***p < 0.001, Two sample *t*‐test.

Figure 5 shows the level of fold resistance to docetaxel, gemcitabine and GEMDOX in the resistant cell lines compared to their respective parental cell line. Fold change was calculated by dividing the IC_50_ value of resistant models by the IC_50_ value of their parental cell line. Across all the resistant sublines of MG‐63, only MG‐63/DOXR8 showed a significant increase of resistance to gemcitabine with 2.44 ± 0.26‐fold (*p* = 0.001). None of the MG‐63 sublines showed a significant change in resistance to docetaxel (Figure 5A). Resistant sublines of HOS‐143B were not resistant to gemcitabine. However, HOS‐143B/MTXR8 was significantly resistant to docetaxel with 2.32 ± 0.17‐fold (*p* = 0.005) compared to HOS‐143B (Figure 5B).

#### 3.5.2 Sensitivity profile of GEMDOX treatment combination

Resistant sublines MG‐63/DOXR8 exhibited a significant fold resistant to the combination of gemcitabine and docetaxel with 2.50 ± 0.53‐fold (*p* = 0.04) compared to parental control MG‐63 (Figure 5A). HOS‐143B/MTXR8 and HOS‐143B/TRIR8 were both showing a significant fold resistant to the combination of drugs with 2.09 ± 0.32‐fold (*p* = 0.017) and 2.44 ± 0.41‐fold (*p* = 0.013) respectively comparing to parental control HOS‐143B (Figure 5B).

## 4 DISCUSSION

### 4.1 Systematic review study

#### 4.1.1 Disease control data

The response rate of primary osteosarcoma was previously found to be worse in older patients than younger patients, mainly due to lower drug doses administered due to intolerance of chemotherapy toxicities and the higher prevalence of tumour in axial locations.^2^ In this systematic review study, the GEMDOX combination regimen showed a similar result as a higher disease control rate was found in younger patients (<18) GEMDOX regimen (%) than the in older patients (>18) (47.2% (95% CI 35.7%–58.8%), *p* < 0.05) (Figure 3A). The disease control rate included patients who experienced a CR, a PR, as well as SD.

The doses of gemcitabine and docetaxel recommended by the NHS for the treatment of sarcomas is 675 mg/m^2^ of gemcitabine and 75 mg/m^2^ of docetaxel, with the indication of increasing these doses to 900 and 1000 mg/m^2^ respectively if the standard dose is well tolerated.^34^ The included eligible studies in this systematic review study have used three different doses of gemcitabine in the GEMDOX regimen as shown in Table 2, including the lowest 675, 900 and the highest 1000 mg/m^2^. There was no significant difference in the disease control rate between the doses of gemcitabine used in the GEMDOX regimen (Figure 3B). This suggests that the doses of gemcitabine used at 675 mg/m^2^ contributed the same efficacy as the doses at 1000 mg/m^2^. Therefore, patients may benefit more from the gemcitabine doses at 675 mg/m^2^ to reduce the likelihood of toxicity or adverse events.

#### 4.1.2 Toxicity data

The most common adverse effects from the GEMDOX regimen are haematological toxicities including neutropenia, thrombocytopenia and anaemia.  Ferrari et al. showed that in non‐metastatic osteosarcoma children below the age of 14 years and female patients experienced a higher incidence of grade 4 thrombocytopenia and neutropenia with MAP + high‐dose ifosfamide.^35^ In contrast, the Chi‐squared analysis performed in this study shows no significant association between the incidences of grade 3 and 4 toxicities (neutropenia, thrombocytopenia and anaemia) reported on GEMDOX regimen and the characteristics of the patients (age and doses of gemcitabine) as shown in Figure 4.

#### 4.1.3 Gender

Previous studies have shown female osteosarcoma patients have a better response rate compared to male patients.^36^, ^37^, ^38^ We were unable to do a quantitative analysis of the role gender in the response to GEMDOX as the included studies did not break down the number of male and female responders. However, we were able to observe an interesting trend. Studies that had a higher proportion of male patients^25^, ^26^, ^27^ had a higher response rate to GEMDOX, than those who had a balanced gender distribution.^11^, ^33^ Suggesting that male patients may have a better response to GEMDOX than females. In contrast, no difference in toxicities was observed between the studies with a higher proportion of males^25^, ^26^, ^27^ compared to those with a balanced gender distribution.^11^, ^33^

#### 4.1.4 Heterogeneity

A systematic review of non‐randomised studies may observe more heterogeneity than one of randomised controlled trials.^39^ As such, we wish to demonstrate the homogenous nature of our included patient population. 93% of the histology of included relapsed osteosarcoma patients had conventional osteosarcoma (Table 1) and was consistent across studies.^25^, ^31^ This high percentage of conventional osteosarcoma population represents the general population of osteosarcoma patients where it is the most common type of histology (80%).^39^ The total number of male and female patients included in this review was 67 males and 45 females (Table 1), calculated ratio as 1.48:1. The incidence rate ratio of male to female in a general population of osteosarcoma patients is 1.5:1,^40^ which matched with our ratio in this review where male is higher than female. In addition, only patients who had received standard MAP therapy as their first‐line chemotherapy treatment and had not received gemcitabine and docetaxel therapy are included in this review.

#### 4.1.5 In vitro study

Developed osteosarcoma resistant sublines from MG‐63 and HOS‐143B by single‐agent and multi‐agent were used in this study to simulate the similar clinical condition where relapsed osteosarcoma patients received the standard MAP chemotherapy regimen. The acid phosphatase assay was used in determining the cytotoxicity, as the acid phosphatase substrate was the least likely to be effluxed by all the transporters which also transport the drugs being studied.^41^ Only one out of the eight osteosarcoma resistant sublines were resistant to single‐agent docetaxel and gemcitabine treatment (Figure 5A,B). Only three out of eight of the osteosarcoma resistant sublines were determined to have significant fold resistance to GEMDOX combination treatment (Figure 5A,B). This suggests that most of the developed resistant sublines remained comparably sensitive to the combination of gemcitabine and docetaxel compared to their parental control MG‐63 and HOS‐143B. Therefore, this result also indicates patients who acquire drug resistance to the combination treatment of cisplatin, doxorubicin and methotrexate, will have a high potential to remain sensitive to the combination treatment of gemcitabine and docetaxel especially with cisplatin resistance, as MG‐63/CISR8 and HOS‐143B/CISR8 were both not showing resistance to the GEMDOX combination.

The sensitivity to gemcitabine and docetaxel in cisplatin resistant sublines could be due to two possible reasons: (1) different targets of drugs and (2) independent mechanisms of resistance. Cisplatin is a platinum‐based drug that causes DNA damage primarily through the formation of DNA cross‐links, leading to cell death.^42^ However, gemcitabine targets DNA synthesis directly and its efficacy is less likely to be affected by the DNA repair mechanisms that confer resistance to cisplatin.^43^ Similarly, docetaxel disrupts microtubule functions, which is unrelated to the DNA damage and repair pathways affected by cisplatin. Therefore, resistance to cisplatin does not impact the cell's sensitivity to drugs that target the mitotic spindle apparatus.^44^ In addition, gemcitabine and docetaxel also have independent mechanisms of resistance. The primary mechanisms of resistance to gemcitabine would involve alterations in nucleoside transporters or the enzymes involved in its activation,^45^ while resistance to taxanes like docetaxel generally involves alterations in tubulin or the expression of microtubule‐associated proteins.^46^ If these alterations have not occurred in the cisplatin resistant sublines, gemcitabine and docetaxel can still be effective.

On the other hand, there are some common overlapping resistance mechanisms between methotrexate, doxorubicin and gemcitabine, which can provide a possible explanation for the methotrexate and doxorubicin resistant sublines showing significant resistance to gemcitabine. Methotrexate depletes the pool of thymidylate and purine nucleotides by inhibiting DHFR,^47^ and gemcitabine reduces deoxyribonucleotide pools by inhibiting RNR and gets incorporated into DNA.^45^ Methotrexate resistance often involves upregulation of DHFR or reduced drug uptake.^47^ If similar mechanisms confer resistance to nucleotide depletion, they could also reduce gemcitabine efficacy since both nucleotide pools are essential for DNA synthesis. In addition, methotrexate and doxorubicin resistance often involve increased expression of drug efflux pumps (e.g., P‐glycoprotein) and detoxification mechanisms.^48^ These similar efflux pumps or detoxification pathways might also expel or neutralise gemcitabine, contributing to cross‐resistance. While methotrexate, doxorubicin and gemcitabine all disrupt DNA replication and cell proliferation, the specific targets and mechanisms of action differ. Resistance mechanisms developed against methotrexate and doxorubicin, such as enhanced DNA repair, altered drug uptake/efflux and changes in nucleotide metabolism, can confer cross‐resistance to gemcitabine. However, unique aspects of gemcitabine, such as its requirement for activation by dCK and its specific inhibition of RNR,^49^ highlight molecular particularities that can be exploited to overcome resistance in some contexts but also present challenges due to overlapping resistance mechanisms.

## 5 LIMITATIONS

One of the limitations is the lack of complete data from the included studies in the systematic review. Only three out of 11 studies had complete data available for gender, treatment response, survival and toxicity.^25^, ^26^, ^27^ For many studies, the reason that the data was unavailable was because the researchers had included patients with other types of sarcomas, for example, soft tissue or other bone sarcomas in their studies. While combining different disease types make sense when investigating relatively rare cancers, there may be valuable information in the toxicity and survival data for different disease types that is not accessible when the results for all patient groups are combined. The in vitro study showed an insight on the effectiveness of GEMDOX therapy in resistant osteosarcoma cell lines. However, 2‐dimentional cell culture is unable to provide the availability in investigating the disturbance of interactions between cellular and extracellular environments which could be adressed in an in vivo study.^50^

## 6 CONCLUSION

This systematic review study has determined the age of the patients will have a prognostic effect on the GEMDOX regimen as the second‐line treatment for relapsed osteosarcoma. Moreover, the age of patients and the doses used for GEMDOX regimen will not affect the incidence of toxicities. Lastly, most of the osteosarcoma resistant sublines have remained sensitive to either single‐agent gemcitabine, docetaxel and the combination of both, which indicates that the GEMDOX treatment has a high potential efficacy in relapsed osteosarcoma patients especially those with cisplatin resistance.

## AUTHOR CONTRIBUTIONS

**Kaan Low:** Data curation (lead); formal analysis (lead); investigation (lead); writing – original draft (lead). **Paola Foulkes:** Data curation (supporting); formal analysis (supporting); investigation (supporting). **Frank Hills:** Supervision (supporting). **Helen C. Roberts:** Supervision (supporting). **Britta Stordal:** Conceptualization (lead); formal analysis (supporting); methodology (lead); writing – original draft (supporting); writing – review and editing (lead).

## CONFLICT OF INTEREST STATEMENT

The authors declare no conflicts of interest.

## Supporting information


Table S1.

## Data Availability

The data that support the findings of this study are available from the corresponding author upon reasonable request.
