# Supplementary material for: The efficacy of gemcitabine and docetaxel chemotherapy for the treatment of relapsed and refractory osteosarcoma: A systematic review and pre‐clinical study
Source: Cancer Med. 2024 Sep 24;13(18):e70248. doi: 10.1002/cam4.70248 (PMC11420655; doi:10.1002/cam4.70248)
Supplement: Supplementary file 1 — Table S1. [file CAM4-13-e70248-s001.docx]

Table S1 - Result of quality assessment of the 11 studies included in this review.

| **Author/ Year** | **Q1** | **Q2** | **Q2** | **Q4** | **Q5** |
| --- | --- | --- | --- | --- | --- |
| **(Fox *et al.*, 2012)** | Low | Unclear | Unclear | Low | Unclear |
| **(Gosiengfiao *et al.*, 2012)** | Unclear | Low | Low | Low | Unclear |
| **(He *et al.*, 2013)** | Low | Low | Low | Low | Low |
| **(Lee *et al.*, 2016)** | Low | Low | Low | Unclear | Low |
| **(Mora *et al.*, 2009)** | Unclear | Unclear | Low | Low | Unclear |
| **(Navid *et al.*, 2008)** | Low | Low | Low | Low | Unclear |
| **(Palmerini *et al.*, 2016)** | Low | Low | Low | Low | Low |
| **(Rapkin *et al.*, 2012)** | Unclear | Unclear | Low | Low | Low |
| **(Takahashi *et al.*, 2017)** | Unclear | Low | Low | Low | Low |
| **(Xu, Guo and Xie, 2018)** | Low | Low | Low | Low | Low |
| **(Yu *et al.*, 2014)** | Low | Low | Low | Low | Low |
